# Comparison of online health information between different digital platforms for pelvic organ prolapse

**DOI:** 10.1007/s00345-022-04129-6

**Published:** 2022-08-25

**Authors:** Tanja Hüsch, Sita Ober, Axel Haferkamp, Gert Naumann, Ralf Tunn, Matthias Saar, Jennifer Kranz

**Affiliations:** 1 Department of Urology and Pediatric Urology, University Medical Center of Johannes Gutenberg University, Langenbeckstr. 1, 55131 Mainz, Germany; 2 Department of Obstetrics and Gynecology, Hospital Darmstadt, Darmstadt, Germany; 3 Department of Gynecology and Obstetrics, Helios Hospital Erfurt, Erfurt, Germany; 4 Department of Urogynecology, St. Hedwig Hospital, Berlin, Germany; 5 Department of Urology, University Hospital RWTH Aachen, Aachen, Germany; 6 Department of Urology and Kidney Transplantation, Martin-Luther-University, Halle (Saale), Germany

**Keywords:** Social media, Search engine, Pelvic organ prolapse, Communications media, Surgery

## Abstract

**Purpose:**

To identify differences in the content and quality of online health information for pelvic organ prolapse (POP) presented in social media and digital search engines to sustainably enhance patient guidance for adequate platforms for seeking online health information on POP.

**Methods:**

The platforms Google search, Facebook, Instagram, LinkedIn, and YouTube were searched for the keyword “pelvic organ prolapse”. Results were categorized as useful, misleading, advertising, and personal experience. Data were categorized into healthcare professionals, professional organisations, industry, patients, and individuals. The readability score and Health On the Net (HON) code seal were analyzed for Google. Descriptive and univariate analysis was performed.

**Results:**

The source with the highest quantity of useful content was YouTube whereas LinkedIn included mostly advertisement and misleading content. YouTube and Google provided the greatest variety of health information. Social media platforms identified emotional distress and sleep disturbances as a common side effect of POP which is limited considered in clinical practice and provide novel insights of bothersome symptoms related to the disease. The spectrum of different surgical techniques was limited in all platforms. Only 12 (40.0%) were HON-qualified websites with a mean readability score of 10.4 which is considered fairly difficult to read.

**Conclusion:**

Besides Google search, YouTube was identified as a valuable online source for POP information. However, encompassing information of surgical techniques was limited in all platforms. Urogynecological association may contribute to improve patient information by providing online health information which is complete and easy to understand.

**Supplementary Information:**

The online version contains supplementary material available at 10.1007/s00345-022-04129-6.

## Introduction

The “expert patient” is a popular term defining a patient whose knowledge and experience about his chronic disease empowers him to play a part in its management [1]. This trend is mainly based on the availability of online health information, which is becoming increasingly popular among patients [2]. This trend might improve the patients’ involvement in their health and health decision-making [2].

However, concerns about online health information have been raised since the quality of information varies, and the patients’ possibilities for a critical assessment of the information are limited [2]. This may lead to misinformation, distress, increasing tendency for self-diagnosis or self-treatment and may even adversely affect the patient–physician relationship [3].

The online interest in pelvic organ prolapse (POP) has been steadily rising during the last decades [4].

This investigation aimed to identify differences in the quality and content of online health information depending on the utilized source. For this purpose, the online resources Google search, LinkedIn, YouTube, Facebook, and Instagram have been searched for health information about pelvic organ prolapse.

Google is the most popular search engine, and by handling over 3.5 billion searches per day, it is also the most-visited website worldwide [5]. LinkedIn is a business and employment-oriented social network with currently approx. 830 million registered members from 200 countries and territories. The LinkedIn platform enables the possibility to write posts and articles which are shared within the network [6, 7]. YouTube is the largest online video sharing platform. Currently, its users watch more than one billion hours of videos each day. Additionally, it acts as a social network since users may upload their videos, comment, respond and rate to videos and even create playlists or subscribe to other users and channels. It is the second most visited website worldwide, with over one billion monthly users [5, 8]. Facebook is a social media platform and social networking service with more than 2.8 billion monthly active users. Users can post text, photos and multimedia and interact directly with other users [9, 10]. Instagram is a photo and video-sharing social networking service acquired by Facebook in 2021. It has more than one billion users and more than 500 million daily active users [11].

Patients affected by pelvic organ prolapse may search digital health information on its disease prior or during their consultations with physicians. Therefore, the purpose of this investigation was the identification of the content, quantity and quality of online health information which can be found on different digital platforms according to the search term “pelvic organ prolapse”. We furthermore aimed to identify the differences and gaps of information which can be found by the patients on different platforms in order to give guidance on the most appropriate platforms for online health information on pelvic organ prolapse.

## Materials and methods

This research did not require ethical approval since it did not involve human beings or animals. The platforms Google search, Facebook, LinkedIn, Instagram and YouTube were searched between March and June 2021 for the keyword “pelvic organ prolapse”. The web browser's cache and cookies were deleted before the search, and the search was performed in incognito mode.

Firstly, the results were classified into useful [12, 13], misleading, advertising, and personal experience. Useful was defined if the content included scientifically correct information about any aspect of the disease, including, but not limited to, prevention, symptoms, treatment, or pathogenesis. On the contrary, misleading content did not include any information about the target disease, e.g., advertisements, jokes, or job vacancies. Subsequently, the information was categorized by the website's organization into individual health care professionals (HCP), professional associations (i.e., medical school, guideline committee, hospital, etc.), industry, patients, and individuals.

The medical content was analyzed and categorized into pathophysiology, diagnosis, and therapy if applicable. Any treatment option presented at the websites were collected. Furthermore, reported associated diseases with POP were collected accordingly.

The readability score, Alexa Score, and Health On the Net foundation evaluation were analyzed in Google search analytics. The Alexa Score is a global ranking system that sorts websites by popularity. It is calculated based on the estimated average daily number of visitors and page views for a given website in the last three months [14]. The lower the Alexa Rank, the more popular the website. An Alexa rank of 1 million or less is considered to be good.

The Flesch–Kincaid Grade level for readability calculates the effort for reading the text for different levels of education. The higher the score, the more difficult to read the text. The scale has a range from 0 to 18, where 15–18 represents the level of a scientific paper and 0 to 3 learning to read books. Text intended for public readership should aim for a grade level of 8, schooling age 13–14 [15].

The Health on the Net (HON) foundation is a non-governmental organization aiming to ensure quality health information on the internet. HON-qualified internet websites promise verified medical accuracy or correctness and receive a HON code seal if validated [16].

### Statistics

Descriptive analysis has been applied. Univariate and multivariate analyses has been performed to evaluate heterogeneity regarding the distribution of information depending on the source. A *p* value < 0.05 has been considered significant. Statistical analysis was performed by SPSS 26 (IBM, Armonk, United States).

## Results

The first 30 Google search results, presented in three search engine result pages (SERP), were utilized for analysis. This is because about 70% of searchers do not pass the first SERP, including 10 search results, and even 67.6% do not even pass the first five results of the first SERP. The click-through rate in the second and third SERP is only 5.59%, emphasizing the relevance of the listing in the first SERP [17] Therefore, the current search was limited to the first three SERPs, including 30 search results that were applied to all platforms [18]

**Fig. 1:**
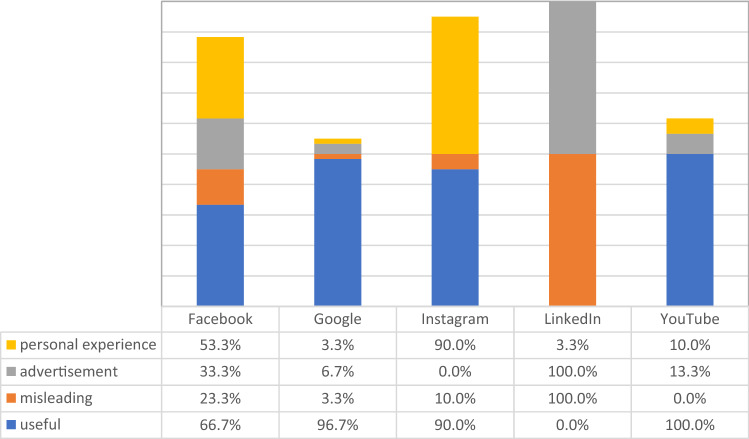
Useful or misleading content, advertisement and personal experience distributed by the source using the keyword “pelvic organ prolapse”

There were significant differences between the source and the occurrence of useful or misleading content *(p* < 0.001), advertisement (*p* < 0.001), and personal experiences (*p* < 0.001). The source with the highest quantity of useful content was YouTube [*n* = 30 (100.0%)]. Advertisement and misleading content were most present in LinkedIn [*n* = 30 (100.0%)] and personal experiences were mostly presented in Instagram [*n* = 27 (90.0%)] (Fig. 1).

There were significant differences in the distribution of organizations between the sources (*p* < 0.001, online resource 3). The most frequent organization in Google search [*n* = 25 (83.3%)], YouTube [*n* = 20 (66.7%)] and LinkedIn [*n* = 9 (30.0%)] were professional organizations. HCP were the most frequent organization in Instagram [*n* = 12 (40.0%)]. Industry and individuals were not present on YouTube. Furthermore, no HCP, individual or patient was present on LinkedIn.

Regarding medical content, pathophysiology was most frequently addressed on YouTube [*n* = 22 (73.3%)], whereas diagnostics and surgical treatment was most frequently addressed on Google. Conservative treatment was most frequently identified in Instagram [*n* = 27 (90.0%), online resource 1); however, it was limited to pelvic floor exercises only.

Information about pathophysiology, diagnostic and treatment options were highly limited or absent in LinkedIn and Instagram despite pelvic floor exercises in Instagram. YouTube and Google provided the highest diversity of medical content.

The most frequently presented treatment option in all sources were pelvic floor exercises (Table 1).

**Table 1:** Treatment options presented in online content distributed by source

Treatment	Source					
	Facebook	Google	Instagram	LinkedIn	YouTube	*P* value
Conservative						
Pessary, *n *(%)	11 (36.7)	22 (73.3)	1 (3.3)	0	11 (36.7)	< 0.001*
Pelvic floor exercises, *n *(%)	18 (60.0)	25 (83.3)	27 (90.0)	1 (3.3)	16 (53.3)	< 0.001*
*Surgical*						
Route, *n *(%)						
Vaginal	9 (30.0)	18 (60.0)	0	0	8 (26.7)	< 0.001*
Abdominal	4 (13.3)	16 (53.3)	0	0	12 (40.0)	< 0.001*
Robotic-assisted surgery, *n *(%)	3 (10.0)	10 (33.3)	0	0	8 (26.7)	< 0.001*
Laparoscopic surgery, *n *(%)	3 (10.0)	15 (50.0)	0	0	11 (36.7)	< 0.001*
Native-tissue vaginal, *n *(%)	0	7 (23.3)	0	0	7 (23.3)	< 0.001*
Native-tissue abdominal, *n *(%)	0	4 (13.3)	0	0	7 (23.3)	< 0.001*
Mesh-augmented surgery, *n *(%)	9 (30.0)	14 (46.7)	0	0	6 (20.0)	< 0.001*
Uterine sparing technique, *n *(%)	4 (13.3)	10 (33.3)	0	0	11 (36.7)	< 0.001*
Sacrocolpopexy, *n *(%)	3 (10.0)	11 (36.7)	0	0	7 (23.3)	< 0.001*
Colpocleisis, *n *(%)	0	15 (50.0)	0	0	3 (10.0)	< 0.001*
Colporrhaphy, *n *(%)	3 (10.0)	15 (50.0)	0	0	6 (20.0)	< 0.001*

^*^Sig. *p* < 0.05

The most frequent reported secondary diseases related to pelvic organ prolapse were birth trauma on Google, YouTube and Instagram followed by faecal incontinence, sexual dysfunction, or sleep disturbances. Overactive bladder (OAB) or stress urinary incontinence (SUI) were reported in up to 36.7% (online resource 2).

In Google search, the mean Alexa score for the Google search POP was 360.039, and 12 (40.0%) had an HON-code seal. The mean readability score was 10.4 (range 7–15), the mean daily page views per visitor were 2.1, and the mean daily time on the sites were 2.14 min.

## Discussion

This investigation evaluated the informational value of pelvic organ prolapse in different digital platforms. In a cross-sectional assessment of POP, useful information was most found on YouTube, followed by a Google search in more than 90%. The content was most frequently provided by professional organizations in both sources. LinkedIn was identified as a source with the highest rate of advertisement, misleading content, and no useful medical information. The source with the highest diversity and quantity of medical content was Google search, followed by YouTube. Interestingly, the most frequently reported treatment option were pelvic floor exercises in all sources. However, a comprehensive overview and inclusion of all available surgical treatment options for POP was highly limited in all sources. Furthermore, the most frequently reported associated diseases with POP were birth trauma, sexual dysfunction, sleep disturbances and fecal incontinence. OAB and SUI were only reported in up to 36.7% in google search.

Women with POP who present at private practices frequently use the internet and social media to inform themselves about their disease [19]. This is confirmed by the low Alexa rank, indicating that the search results are widespread. However, only 40% were HON-qualified, showing less than half of the Google search results were certified for medical accuracy of correctness. In comparison, the HON code seal was available in 1.8–42.9% of the websites searching for other medical diseases [20–23], thus, POP search is localized at the upper end of HON qualified websites in comparison to other diseases. Additionally, the readability score was 10.4, indicating that the content is fairly difficult to read and best understood by people from 10 to 12th grade US educational system. The readability score is challenging for the patient and might lead to misinformation. The evaluation of an acceptable readability score at the point of care in the US identified that the average US resident read at or below 8th grade level. Thus, website content is mostly not appropriate for knowledge transfer in most cases. However, even patient education materials have been identified to be written above 8th grade [24], being considered challenging and misleading for the patients. In conclusion, POP search in google is popular and a relatively large proportion of websites provide certified content. However, the content is difficult to read and understand for the majority of patients which might represent an important source of misinformation.

Surprisingly in this investigation, besides Google search, YouTube was identified as a useful source for health information of POP. YouTube has been identified in a systematic review as an increasing platform for disseminating health information and includes trustworthy and high-quality information [25]. Contradictory, Herbert et al. [26] evaluated in-depth the YouTube content for POP and pointed out that half of the videos had moderate to poor understandability and many videos were low quality. However, considering the high number of HCO and HCP providing the POP content and the diversity of medical content of the videos, there is a remarkable source of information that YouTube can derive. An increasing trend of helpful information found in social media and an increased number of health professionals using social media themselves have already been identified [13].

In comparison to YouTube and Google search, Facebook provides significantly less information regarding useful medical content for POP. Most of the information for POP was provided by professional associations, disseminating predominantly pathophysiology and conservative treatment information. In a cross-sectional study from 2015, the number of youths still interested in health information on Facebook was 72.8%. Furthermore, half of the participants classified Facebook health-related information as useful and even appr. 11% would follow health advice from Facebook [27]. However, digital trends may vary enormously several years later, and novel insights are lacking.

Interestingly, the most frequent reported diseases correlated to POP were birth trauma and sexual dysfunction. Furthermore, sleep disturbances and emotional distress were reported between 40 and 56.7% whereas OAB symptoms or SUI was reported in only up to 36.7%. Social media might give novel insights into patient perceptions since sleep disturbances or emotional distress are not commonly reported symptoms in POP [28] or in focus of clinical daily practice when treating women for POP. Social media might provide a reliable source on patient perceptions and needs which has not been identified previously by a pure medical perspective. These findings have been confirmed by a recent investigation, identifying sleep disturbances by half of the women with POP [29] thus, representing a common, unrecognized health issue of the patients.

Regarding the variety of treatment of POP, it could be demonstrated that there is still a lack of comprehensive summaries of treatment options and pathophysiology. Even in Google search, there was a considerable discrepancy of available reported surgical techniques for POP. Although commercial businesses can provide helpful information, pharmaceutical or medical device industry-related content was sparse. This emphasizes the important role the physicians in this context play, since the online medical information is not complete and selective. The physician must not only be aware of the variety of treatment options but also has to put the information into the correct context. Depending on the informational sources the patient has utilized, it might be challenging to adjust the patient’s perception.

Besides, LinkedIn and Instagram have been identified as a non-reliable source for seeking health information of POP. Instagram has been only identified as a source for widespread of pelvic floor exercises.

We acknowledge a few limitations of this investigation. Searches were limited to the first 30 results of each platform. However, as previously described, the majority of persons will not consider any search results after even the first 10 results. Therefore, the current results are representative for daily practice. Other social media platforms such as Twitter have not been analyzed and are focus of future research. However, this investigation gives a comprehensive and valuable overview of medical content for different online platforms where patients seeking online health information.

In conclusion, there is an increasing number of useful health information for POP with a steadily rising interest of patients seeking digital health information. However, the readability of health information in Google search was limited to higher education and may limit the accessibility for patients. Most Google search results still lack certified medical accuracy and correctness by HON code seal. Furthermore, LinkedIn and Instagram are non-reliable sources for health information of POP and provide almost no qualitative information at all. Furthermore, social media platforms identified and emphasized different aspects of pelvic organ prolapse, such as emotional distress and sexual dysfunction which are often neglected in clinical daily practice.

Importantly, a lack of adequate comprehensive content covering all aspects of POP repair has been identified for all sources. This emphasizes the crucial role of the physicians providing comprehensive information, putting the patients’ needs and symptoms into context und providing individualized treatment alternatives. Urogynecological associations should consider providing online health information which is easy to read and complete to address patients need.

## Supplementary Information

Below is the link to the electronic supplementary material.

Supplementary file1 (DOCX 28 KB)

Supplementary file2 (DOCX 16 KB)

Supplementary file3 (DOCX 29 KB)
