# Supplementary material for: Comparison of online health information between different digital platforms for pelvic organ prolapse
Source: World J Urol. 2022 Aug 25;40(10):2529–34. doi: 10.1007/s00345-022-04129-6 (PMC9512708; doi:10.1007/s00345-022-04129-6)
Supplement: Supplementary file 1 — Supplementary file1 (DOCX 28 KB) [file 345_2022_4129_MOESM1_ESM.docx]

Figure 3 Medical content distributed by source using the keyword “pelvic organ prolapse”
