# Supplementary material for: Comparison of online health information between different digital platforms for pelvic organ prolapse
Source: World J Urol. 2022 Aug 25;40(10):2529–34. doi: 10.1007/s00345-022-04129-6 (PMC9512708; doi:10.1007/s00345-022-04129-6)
Supplement: Supplementary file 2 — Supplementary file2 (DOCX 16 KB) [file 345_2022_4129_MOESM2_ESM.docx]

Online Resource 2 Reported associated diseases distributed by source

| Associated diseases | Facebook | Google | Instagram | LinkedIn | YouTube | p-value |
| --- | --- | --- | --- | --- | --- | --- |
| Overactive bladder, *n (%)* | 1 (3.3) | 11 (36.7) | 3 (10.0) | 0 | 0 | <0.001* |
| Stress urinary incontinence, *n (%)* | 1 (3.3) | 11 (36.7) | 6 (20.0) | 0 | 0 | <0.001* |
| Mixed urinary incontinence, *n (%)* | 0 | 12 (40.0) | 0 | 0 | 0 | <0.001* |
| Feacal incontinence, *n (%)* | 1 (3.3) | 15 (50.0) | 1 (3.3) | 0 | 0 | <0.001* |
| Sexual dysfunction, *n (%)* | 1 (3.3) | 12 (40.0) | 8 (26.7) | 0 | 16 (53.3) | <0.001* |
| Birth trauma, *n (%)* | 2 (6.7) | 22 (73.3) | 17 (56.7) | 0 | 21 (70.0) | <0.001* |
| Sleep disturbances/emotional stress, *n (%)* | 9 (30.0) | 12 (40.0) | 0 | 0 | 17 (56.7) | <0.001* |
| Other/non-specific, *n (%)* | 11 (36.7) | 0 | 6 (20.0) | 0 | 0 | <0.001* |

*sig. p<0.05
