# Supplementary material for: Comparison of online health information between different digital platforms for pelvic organ prolapse
Source: World J Urol. 2022 Aug 25;40(10):2529–34. doi: 10.1007/s00345-022-04129-6 (PMC9512708; doi:10.1007/s00345-022-04129-6)
Supplement: Supplementary file 3 — Supplementary file3 (DOCX 29 KB) [file 345_2022_4129_MOESM3_ESM.docx]

Online resource 3 Organization distributed by the source using the keyword “pelvic organ prolapse”
